# Computing the skewness of the phylogenetic mean pairwise distance in linear time

**DOI:** 10.1186/1748-7188-9-15

**Published:** 2014-06-14

**Authors:** Constantinos Tsirogiannis, Brody Sandel

**Affiliations:** 1 MADALGO, Center for Massive Data Algorithmics, a Center of the Danish National Research Foundation, Aarhus University, Aarhus, Denmark; 2 Department of Bioscience, Aarhus University, Aarhus, Denmark

**Keywords:** Algorithms for phylogenetic trees, Mean pairwise distance, Skewness

## Abstract

**Background:**

The phylogenetic Mean Pairwise Distance (MPD) is one of the most popular measures for computing the phylogenetic distance between a given group of species. More specifically, for a phylogenetic tree  and for a set of species *R* represented by a subset of the leaf nodes of , the MPD of *R* is equal to the average cost of all possible simple paths in  that connect pairs of nodes in *R*.

Among other phylogenetic measures, the MPD is used as a tool for deciding if the species of a given group *R* are closely related. To do this, it is important to compute not only the value of the MPD for this group but also the expectation, the variance, and the skewness of this metric. Although efficient algorithms have been developed for computing the expectation and the variance the MPD, there has been no approach so far for computing the skewness of this measure.

**Results:**

In the present work we describe how to compute the skewness of the MPD on a tree  optimally, in *Θ*(*n*) time; here *n* is the size of the tree . So far this is the first result that leads to an exact, let alone efficient, computation of the skewness for any popular phylogenetic distance measure. Moreover, we show how we can compute in *Θ*(*n*) time several interesting quantities in , that can be possibly used as building blocks for computing efficiently the skewness of other phylogenetic measures.

**Conclusions:**

The optimal computation of the skewness of the MPD that is outlined in this work provides one more tool for studying the phylogenetic relatedness of species in large phylogenetic trees. Until now this has been infeasible, given that traditional techniques for computing the skewness are inefficient and based on inexact resampling.

## Background

Communities of co-occuring species may be described as “clustered” if species in the community tend to be close phylogenetic relatives of one another, or “overdispersed” if they are distant relatives [1]. To define these terms we need a function that measures the phylogenetic relatedness of a set of species, and also a point of reference for how this function should behave in the absence of ecological and evolutionary processes. One such function is the mean pairwise distance (MPD); given a phylogenetic tree  and a subset of species *R* that are represented by leaf nodes of , the MPD of the species in *R* is equal to average cost of all possible simple paths that connect pairs of nodes in *R*.

To decide if the value of the MPD for a specific set of species *R* is large or small, we need to know the average value (expectation) of the MPD for all sets of species in  that consist of exactly *r*=|*R*| species. To judge how much larger or smaller is this value from the average, we also need to know the standard deviation of the MPD for all possible sets of *r* species in . Putting all these values together, we get the following index that expresses how clustered are the species in *R*[1]:

$$\text{NRI}=\frac{\text{MPD}(T,R)-{\text{expec}}_{\text{MPD}}(T,r)}{{\text{sd}}_{\text{MPD}}(T,r)},$$

where MPD(T,R) is the value of the MPD for *R* in , and expec(T) and ${\text{sd}}_{\text{MPD}}(T,r)$ are the expected value and the standard deviation respectively of the MPD calculated over all subsets of *r* species in .

In a previous paper we presented optimal algorithms for computing the expectation and the standard deviation of the MPD of a phylogenetic tree  in *O*(*n*) time, where *n* is the number of the edges of [2]. This enabled exact computations of these statistical moments of the MPD on large trees, which were previously infeasible using traditional slow and inexact resampling techniques. However, an important problem remained unsolved; quantifying our degree of confidence that the NRI value observed in a community reflects non-random ecological and evolutionary processes.

This degree of confidence can be expressed as a statistical *P* value, that is the probability that we would observe an NRI value as extreme or more so if the community was randomly assembled. Traditionally, estimating *P* is accomplished by ranking the observed MPD against the distribution of randomized MPD values [3]. If the MPD falls far enough into one of the tails of the distribution (generally below the 2.5 percentile or above the 97.5 percentile, yielding *P*<0.05), the community is said to be significantly overdispersed or significantly clustered. However, this approach relies on sampling a large number of random subsets of species in , and recomputing the MPD for each random subset. Therefore, this method is slow and imprecise. This problem is exacerbated when it is necessary to consider multiple trees at once, arising for example from a Bayesian posterior sample of trees [4,5]. In such cases, sufficient resampling from all trees in the sample can be computationally limiting.

We can approximate the *P* value of an observed NRI by assuming a particular distribution of the possible MPD values and evaluating its cumulative distribution function at the observed MPD. Because the NRI measures the difference between the observed values and expectation in units of standard deviations, this yields a very simple rule if we assume that possible MPD values are normally distributed: any NRI value larger than 1.96 or smaller than -1.96 is significant. Unfortunately, the distribution of MPD values is often skewed, such that this simple rule will lead to incorrect *P* value estimates [6,7]. Of particular concern, this skewness introduces a bias towards detecting either significant clustering or significant overdispersion [8]. If the distribution of MPD values for a particular tree can be reasonably approximated using a skew-normal distribution, calculating the skewness analytically would enable us to remove this bias and improve the accuracy of *P* value estimates. In the last part of the paper, we describe experiments on large randomly generated trees, supporting this argument. Further, when a large sample of trees should be considered, the full distribution of MPD values can be considered as a mixture of skew-normal distributions [9,10], greatly simplifying and speeding up the process of calculating *P* values across the entire set of trees.

However, so far there has been no result in the related literature that shows how to compute the needed skewness measure efficiently. Hence, given a phylogenetic tree  and an integer *r* there is the need to design an efficient and exact algorithm that can compute the skewness of the MPD for *r* species in . This would provide the last critical piece required for the adoption of a fully analytical and efficient approach for analysing ecological communities using the MPD and the NRI.

### Our results

In the present work we show how we can compute efficiently the skewness of the MPD. More specifically, given a tree  that consists of *n* edges and a positive integer *r*, we prove that we can compute the skewness of of the MPD over all subsets of *r* leaf nodes in  optimally, in *Θ*(*n*) time. For the calculation of this skewness value we consider that every subset of exactly *r* species in  is picked uniform with probability out of all possible subsets that have *r* species. The main contribution of this paper is a constructive proof that leads straightforwardly to an algorithm that computes the skewness of the MPD in *Θ*(*n*) time. This is clearly optimal, and it outperforms even the best algorithms that are known so far for computing lower-order statistics for other phylogenetic measures; for example the best known algorithm for computing the variance of the popular Phylogenetic Diversity (PD) runs in *O*(*n*^2^) time [2].

More than that, we prove how we can compute in *Θ*(*n*) time several quantities that are related with groups of paths in the given tree; these quantities can be possibly used as building blocks for computing efficiently the skewness (and other statistical moments) of phylogenetic measures that are similar to the MPD. Such an example is the measure which is the equivalent of the MPD for computing the distance between two subsets of species in [11].

The rest of this paper is, almost in its entirety, an elaborate proof for computing the skewness of the MPD on a tree  in *Θ*(*n*) time. In the next section we define the problem that we want to tackle, and we present a group of quantities that we use as building blocks for computing the skewness of the MPD. We prove that all of these quantities can be computed in linear time with respect to the size of the input tree. Then, we provide the main proof of this paper; there we show how we can express the value of the skewness of the MPD in terms of the quantities that we introduced earlier. The proof implies a straightforward linear time algorithm for the computation of the skewness as well. In the last section we provide experimental results that indicate that computing the skewness of the MPD can be a useful tool for improving the estimation of *P* values when a skew-normal distibution is assumed. There we describe experiments that we conducted on large randomly generated trees to compare two different methods for estimating *P* values; one method is based on random sampling of a large number of tip sets, and the other method relies in calculating the mean, variance, and skewness of the MPD for the given tree.

## Description of the problem and basic concepts

### Definitions and notation

Let  be a phylogenetic tree, and let *E* be the set of its edges. We denote the number of the edges in  by *n*, that is *n*=|*E*|. For an edge *e*∈*E*, we use *w*_*e*_ to indicate the weight of this edge. We use *S* to denote the set of the leaf nodes of . We call these nodes the *tips* of the tree, and we use *s* to denote the number of these nodes.

Since a phylogenetic tree is a rooted tree, for any edge *e*∈*E* we distinguish the two nodes adjacent to *e* into a *parent* node and a *child* node; among these two, the parent node of *e* is the one for which the simple path from this node to the root does not contain *e*. We use Ch(*e*) to indicate the set of edges whose parent node is the child node of *e*, which of course implies that *e*∉Ch(*e*). We indicate the edge whose child node is the parent node of *e* by parent(*e*). For any edge *e*∈*E*, tree T(e) is the subtree of  whose root is the child node of edge *e*. We denote the set of tips that appear in T(e) as *S*(*e*), and we denote the number of these tips by *s*(*e*).

Given any edge *e*∈*E*, we partition the edges of  into three subsets. The first subset consists of all the edges that appear in the subtree of *e*. We denote this set by Off(*e*). The second subset consists of all edges *e*^′^∈*E* for which *e* appears in the subtree of *e*^′^. We use Anc(*e*) to indicate this subset. For the rest of this paper, we define that *e*∈Anc(*e*), and that *e*∉Off(*e*). The third subset contains all the tree edges that do not appear neither in Off(*e*), nor in Anc(*e*); we indicate this subset by Ind(*e*).

For any two tips *u*,*v*∈*S*, we use *p*(*u*,*v*) to indicate the simple path in  between these nodes. Of course, the path *p*(*u*,*v*) is unique since  is a tree. We use *cost*(*u*,*v*) to denote the cost of this path, that is the sum of the weights of all the edges that appear on the path. Let *u* be a tip in *S* and let *e* be an edge in *E*. We use *cost*(*u*,*e*) to represent the cost of the shortest simple path between *u* and the child node of *e*. Therefore, if *u*∈*S*(*e*) this path does not include *e*, otherwise it does. For any subset *R*⊆*S* of the tips of the tree , we denote the set of all pairs of elements in *R*, that is the set of all combinations that consist of two distinct tips in *R*, by *Δ*(*R*). Given a phylogenetic tree  and a subset of its tips *R*⊆*S*, we denote the Mean Pairwise Distance of *R* in  by MPD(T,R). Let *r*=|*R*|. This measure is equal to:

$$\begin{array}{l} \text{MPD}(T,R)=\frac{2}{r(r-1)}\underset{\left\{u,v\}\in \Delta (R\right)}{\sum }\text{cost}(u,v)\;. \end{array}$$

### Aggregating the costs of paths

Let  be a phylogenetic tree that consists of *n* edges and *s* tips, and let *r* be a positive integer such that *r*≤*s*. We use sk(T,r) to denote the skewness of the MPD on  when we pick a subset of *r* tips of this tree with uniform probability. In the rest of this paper we describe in detail how we can compute sk(T,r) in *O*(*n*) time, by scanning  only a constant number of times. Based on the formal definition of skewness, the value of sk(T,r) is equal to:

(1) $$\;\begin{array}{l} \text{sk}(T,r)=E_{R\in \text{Sub}(S,r)}\left[{\left(\frac{\text{MPD}(T,R)-\text{expec}(T,r)}{\text{var}(T,r)}\right)}^{3}\right]\\ =\frac{E_{R\in \text{Sub}(S,r)}\left[{\text{MPD}}^{3}(T,R)\right]-3\cdot \text{var}{\left(T,r\right)}^{2}-\text{expec}{\left(T,r\right)}^{3}}{\text{var}{\left(T,r\right)}^{3}}\;, \end{array}$$

where expec(T,r) and var(T,r) are the expectation and the variance of the MPD for subsets of exactly *r* tips in , and *E*_*R*∈Sub(*S*,*r*)_[·] denotes the function of the expectation over all subsets of exactly *r* tips in *S*. In a previous paper, we showed how we can compute the expectation and the variance of the MPD on  in *O*(*n*) time [2]. Therefore, in the rest of this work we focus on analysing the value $E_{R\in \text{Sub}(S,r)}[{\text{MPD}}^{3}(T,R)]$ and expressing this quantity in a way that can be computed efficiently, in linear time with respect to the size of .

To make things more simple, we break the description of our approach into two parts; in the first part, we define several quantities that come from adding and multiplying the costs of specific subsets of paths between tips of the tree. We also present how we can compute all these quantities in *O*(*n*) time in total by scanning  a constant number of times. Then, in the next section, we show how we can express the skewness of the MPD on  based on these quantities, and hence compute the skewness in *O*(*n*) time as well. Next we provide the quantities that we want to consider in our analysis; these quantities are described in Table 1. In this table but also in the rest of this work, for any tip *u*∈*S*, we consider that SQ(*u*)=SQ(*e*), and TC(*u*)=TC(*e*), such that *e* is the edge whose child node is *u*.

**Table 1:** The quantities that we use for expressing the skewness of the MPD

I) $\text{TC}(T)=\underset{\left\{u,v\}\in \Delta (S\right)}{\sum }\text{cost}(u,v)$	II) $\text{CB}(T)=\underset{\left\{u,v\}\in \Delta (S\right)}{\sum }\text{cos}t^{3}(u,v)$
III) $\forall e\in E,\;\text{TC}(e)=\underset{\begin{array}{c} \left\{u,v\}\in \Delta (S\right)\\ e\in p(u,v) \end{array}}{\sum }\text{cost}(u,v)$	IV) $\forall e\in E,\;\text{SQ}(e)=\underset{\begin{array}{c} \left\{u,v\}\in \Delta (S\right)\\ e\in p(u,v) \end{array}}{\sum }\text{cos}t^{2}(u,v)$
V) $\forall e\in E,\;\text{Mult}(e)=\underset{\begin{array}{c} \left\{u,v\}\in \Delta (S\right)\\ e\in p(u,v) \end{array}}{\sum }\text{TC}(u)\cdot \text{TC}(v)$	VI) $\forall u\in S,\;\text{SM}(u)=\underset{v\in S\setminus \{u\}}{\sum }\text{cost}(u,v)\cdot \text{TC}(v)$
VII) $\forall e\in E,\;{\text{TC}}_{\text{sub}}(e)=\underset{\begin{array}{c} u\in S(e) \end{array}}{\sum }\text{cost}(u,e)$	VIII) $\forall e\in E,\;{\text{SQ}}_{\text{sub}}(e)=\underset{\begin{array}{c} u\in S(e) \end{array}}{\sum }\text{cos}t^{2}(u,e)$
IX) $\forall e\in E,\;\text{PC}(e)=\underset{\begin{array}{c} u\in S \end{array}}{\sum }\text{cost}(u,e)$	X) $\forall e\in E,\;\text{PSQ}(e)=\underset{\begin{array}{c} u\in S \end{array}}{\sum }\text{cos}t^{2}(u,e)$
XI) $\forall e\in E,\;\text{QD}(e)=\underset{u\in S(e)}{\sum }{\left(\underset{v\in S(e)\setminus \{u\}}{\sum }\text{cost}(u,v)\right)}^{2}$	

We provide now the following lemma.

#### Lemma 1

Given a phylogenetic tree  that consists of *n* edges, we can compute all the quantities that are presented in Table 1 in *O*(*n*) time in total.

#### *Proof*.

Each of the quantities (I)-(X) in Table 1 can be computed by scanning a constant number of times the input tree , either bottom-up or top-to-bottom. For computing quantity (XI) we follow a more involved divide-and-conquer approach.

We showed in a previous paper how we can compute quantity (I) and the quantities in (III) for all *e*∈*E* in *O*(*n*) time in total [2].

For an edge *e*∈*E*, the quantity in (VII) can be written as:

$$\begin{array}{lll} & {\text{TC}}_{\text{sub}}(e)=\underset{u\in S(e)}{\sum }\text{cost}(u,e)=\underset{l\in \text{Off}(e)}{\sum }w_{l}\cdot s(l)\;.\; & \; \end{array}$$

We can compute this quantity for every *e*∈*E* in linear time as follows; in the first scan we compute for every edge *e* the number of leaves *s*(*e*) in T(e). This can be done in *O*(*n*) time by computing in a bottom-up manner *s*(*e*) as the sum of the numbers of tips *s*(*e*^′^), ∀*e*^′^∈Ch(*e*). Then, we can compute *TC*_*s* *u* *b*_(*e*) by scanning bottom-up the tree using the following formula:

$$\begin{array}{ll} & {\text{TC}}_{\text{sub}}(e)=\underset{l\in \text{Ch}(e)}{\sum }w_{l}\cdot s(l)+{\text{TC}}_{\text{sub}}(l)\;.\; \end{array}$$

For quantity (VIII), for any *e*∈*E* we have that:

$$\begin{array}{ll} \;{\text{SQ}}_{\text{sub}}(e) & =\underset{u\in S(e)}{\sum }\text{cos}t^{2}(u,e)\;\\ & =\underset{l\in \text{Off}(e)}{\sum }w_{l}\underset{k\in \text{Off}(l)}{\sum }2\cdot w_{k}\cdot s(k)+\underset{l\in \text{Off}(e)}{\sum }w_{l}^{2}\cdot s(l)\;\\ & =\underset{l\in \text{Off}(e)}{\sum }2\cdot w_{l}\cdot {\text{TC}}_{\text{sub}}(l)+w_{l}^{2}\cdot s(l)\;.\; \end{array}$$

Then *SQ*_*s* *u* *b*_(*e*) can be computed for every edge *e*∈*E* by scanning  bottom up and evaluating the formula:

$$\begin{array}{ll} \; & {\text{SQ}}_{\text{sub}}(e)=\underset{l\in \text{Ch}(e)}{\sum }2\cdot w_{l}\cdot {\text{TC}}_{\text{sub}}(l)+w_{l}^{2}\cdot s(l)+{\text{SQ}}_{\text{sub}}(l)\;.\; \end{array}$$

For every edge *e* in , quantity (IV) can be written as:

$$\begin{array}{ll} \underset{\begin{array}{c} \left\{u,v\}\in \Delta (S\right)\\ e\in p(u,v) \end{array}}{\sum }\text{cos}t^{2}(u,v)= & \;2\underset{l,k\in E}{\sum }w_{l}\cdot w_{k}\cdot \text{NumPath}(e,l,k)\;\\ & +\underset{l\in E}{\sum }w_{l}^{2}\cdot \text{NumPath}(e,l)\;.\; \end{array}$$

In the last expression, value NumPath(*e*,*l*,*k*) is equal to the number of simple paths that connect two tips in  and which also contain all three edges *e*,*l* and *k*. The quantity NumPath(*e*,*l*) is equal to the number of simple paths that connect two tips in  and which also contain both edges *e* and *l*. Therefore, for any *e*∈*E* we have:

(2) $$\;\begin{array}{l} \underset{\begin{array}{c} \left\{u,v\}\in \Delta (S\right)\\ e\in p(u,v) \end{array}}{\sum }\text{cos}t^{2}(u,v)=2(s-s(e))\underset{l\in \text{Off}(e)}{\sum }w_{l}\underset{k\in \text{Off}(l)}{\sum }w_{k}\cdot s(k)\\ \;\;+2\underset{l\in \text{Anc}(e)}{\sum }w_{l}(s-s(l))\underset{k\in \text{Off}(e)}{\sum }w_{k}\cdot s(k)\\ \;\;+2\cdot s(e)\underset{l\in \text{Anc}(e)}{\sum }w_{l}(s-s(l))\underset{\begin{array}{c} k\in \text{Anc}(e)\\ k\in \text{Off}(l) \end{array}}{\sum }w_{k}\\ \;\;+2\cdot s(e)\underset{l\in \text{Ind}(e)}{\sum }w_{l}\underset{k\in \text{Off}(l)}{\sum }w_{k}\cdot s(k)\\ \;\;+2\underset{l\in \text{Ind}(e)}{\sum }w_{l}\cdot s(l)\underset{k\in \text{Off}(e)}{\sum }w_{k}\cdot s(k)\\ \;\;+2\cdot s(e)\underset{l\in \text{Anc}(e)}{\sum }w_{l}\underset{k\in \text{Ind}(l)}{\sum }w_{k}\cdot s(k)\\ \;\;+(s-s(e))\underset{l\in \text{Off}(e)}{\sum }w_{l}^{2}\cdot s_{l}+s(e)\\ \;\;\underset{l\in \text{Anc}(e)}{\sum }w_{l}^{2}\;\cdot \;(s-s(l))\;+\;s(e)\underset{l\in \text{Ind}(e)}{\sum }w_{l}^{2}\;\cdot \;s(l)\\ \;=(s-s(e))\cdot {\text{SQ}}_{\text{sub}}(e)\\ \;\;+\;\;\underset{l\in \text{Anc}(e)}{\sum }\;w_{l}(s\;-\;s(l)\;)(2\;\cdot \;{\text{TC}}_{\text{sub}}(e)\;+\;w_{l}\;\cdot \;s(e)\;)\\ \;\;+2\cdot s(e)\underset{l\in \text{Anc}(e)}{\sum }w_{l}(s\;-\;s(l))\;\;\left(\;\underset{\begin{array}{c} k\in \text{Anc}(e)\\ k\in \text{Off}(l) \end{array}}{\sum }w_{k}\;\right)\\ \;\;+s(e)\underset{l\in \text{Ind}(e)}{\sum }w_{l}(2\cdot {\text{TC}}_{\text{sub}}(l)\\ \;\;+w_{l}\cdot s(l))\;+\;2\cdot {\text{TC}}_{\text{sub}}(e)\underset{l\in \text{Ind}(e)}{\sum }w_{l}\cdot s(l)\\ \;\;+2\cdot s(e)\underset{l\in \text{Anc}(e)}{\sum }w_{l}\underset{k\in \text{Ind}(l)}{\sum }w_{k}\cdot s(k)\;. \end{array}$$

We explain now how we can compute the six quantities in (2) in *O*(*n*) time, assuming that we have already computed T*C*_*s* *u* *b*_(*e*) and *s*(*e*) for every *e*∈*E*. To make the description simpler, we show in detail how we can compute the second and fourth quantities that appear in the last expression; it is easy to show that the rest of the quantities in (2) can be calculated in a similar manner.

For any *e*∈*E*, we denote the second quantity as follows:

$$\begin{array}{l} \;{\text{SUM}}_{1}(e)=\underset{l\in \text{Anc}(e)}{\sum }w_{l}(s-s(l))(2\cdot TC_{\text{sub}}(e)+w_{l}\cdot s(e))\;. \end{array}$$

We also define the following quantities:

$$\begin{array}{l} {\text{SUM}}_{1A}(e)=\underset{l\in \text{Anc}(e)}{\sum }w_{l}(s-s(l))\;, \end{array}$$

and

$$\begin{array}{l} {\text{SUM}}_{1B}(e)=\underset{l\in \text{Anc}(e)}{\sum }w_{l}^{2}(s-s(l))\;. \end{array}$$

We can calculate SUM_1_(*e*) for every edge *e* by traversing the tree top-to-bottom and evaluating the following expressions:

$$\begin{array}{lll} & \;{\text{SUM}}_{1A}(e)=w_{e}(s-s(e))+{\text{SUM}}_{1A}(\text{parent}(e))\;.\; & \;\\ & \;{\text{SUM}}_{1B}(e)=w_{e}^{2}(s-s(e))+{\text{SUM}}_{1B}(\text{parent}(e))\;.\; & \;\\ & \;{\text{SUM}}_{1}(e)=2\cdot {\text{TC}}_{\text{sub}}(e)\cdot {\text{SUM}}_{1A}(e)+{\text{SUM}}_{1B}(e)\cdot s(e)\;.\; & \; \end{array}$$

To compute the fourth quantity in (2), we use the following quantity:

$${\text{SUM}}_{2}\left(e\right)=\underset{l\in \text{Off}\left(e\right)}{\sum }w_{l}\left(2,\cdot ,{\text{TC}}_{\text{sub}},\left(l\right),+,w_{l},\cdot ,s,\left(l\right)\right)\;.$$

This quantity can be evaluated in *O*(*n*) time for every *e*∈*E* with a bottom-up scan of the tree. We also consider the following value which we can precompute in *O*(*n*) time:

$${\text{SUM}}_{2}\left(T\right)=\underset{e\in E}{\sum }w_{e}\left(2,\cdot ,{\text{TC}}_{\text{sub}},\left(e\right),+,w_{e},\cdot ,s,\left(e\right)\right)\;.$$

For every edge *e*∈*E* we calculate in a top-to-bottom manner the formula:

$$\;{\text{SUM}}_{3}\left(e\right)=w_{e}\left(2,\cdot ,{\text{TC}}_{\text{sub}},\left(e\right),+,w_{e},\cdot ,s,\left(e\right)\right)+{\text{SUM}}_{3}\left(\text{parent},\left(e\right)\right)\;.$$

Then for each tree edge *e*, the fourth quantity in (2) can be computed in constant time as follows:

$$\begin{array}{lll} & s(e)\underset{l\in \text{Ind}(e)}{\sum }w_{l}(2\cdot {\text{TC}}_{\text{sub}}(l)+w_{l}\cdot s(l))\; & \;\\ & =s(e)\cdot ({\text{SUM}}_{2}(T)-{\text{SUM}}_{2}(e)-{\text{SUM}}_{3}(e))\;.\; & \; \end{array}$$

The remaining quantities in (2) can be computed in a quite similar manner as the two quantities that we already described.

Quantity (II) in Table 1 is equal to:

$$\begin{array}{ll} \text{CB}(T) & =\underset{\left\{u,v\}\in \Delta (S\right)}{\sum }\text{cos}t^{3}(u,v)\;\\ & =\underset{e\in E}{\sum }w_{e}\underset{\begin{array}{c} \left\{u,v\}\in \Delta (S\right)\\ e\in p(u,v) \end{array}}{\sum }\text{cos}t^{2}(u,v)=\underset{e\in E}{\sum }w_{e}\cdot \text{SQ}(e)\;.\; \end{array}$$

We have already presented how to compute SQ(*e*) for every edge *e* in  in *O*(*n*) time in total, hence we can also compute CB(T) in *O*(*n*) time by simply summing up the values *w*_*e*_·SQ(*e*) for every edge *e* in the tree. For quantity (V) it holds that:

$$\begin{array}{ll} \;\text{Mult}(e) & =\underset{\begin{array}{c} \left\{u,v\}\in \Delta (S\right)\\ e\in p(u,v) \end{array}}{\sum }\text{TC}(u)\cdot \text{TC}(v)\;\\ & =\underset{u\in S(e)}{\sum }\text{TC}(u)\underset{v\in S-S(e)}{\sum }\text{TC}(v)\;\\ & =\left(\underset{u\in S(e)}{\sum }\text{TC}\left(u\right)\right)\;\left(\underset{v\in S}{\sum }\text{TC}\left(v\right)-\underset{u\in S\left(e\right)}{\sum }\text{TC}\left(u\right)\right).\; \end{array}$$

Since we have already computed TC(*v*) for every tip *v*∈*S*, we can trivially evaluate $\underset{v\in S}{\sum }\text{TC}\left(v\right)$ in *O*(*n*) time. Hence, to compute quantity (V) it remains now to calculate the values ${\text{SUM}}_{4}(e)=\underset{u\in S(e)}{\sum }\text{TC}(u)$ for every edge *e*∈*E*. We can do this in *O*(*n*) time as follows: at each tip *u*∈*S* we store the value TC(*u*) that we have already computed. Then we scan  bottom-up and we calculate SUM_4_(*e*) by summing up the values SUM_4_(*l*) for all edges *l*∈Ch(*e*).

Let *u* be a tip in *S*, and let *e* be the edge which is adjacent to *u*. Then, quantity (VI) is equal to:

$$\begin{array}{lll} \;\text{SM}(u) & =\underset{v\in S\setminus \{u\}}{\sum }\text{cost}(u,v)\cdot \text{TC}(v)\; & \;\\ & =\underset{l\in \text{Anc}(e)}{\sum }w_{l}\underset{v\in S\setminus S(l)}{\sum }\text{TC}(v)+\underset{l\in \text{Ind}(e)}{\sum }w_{l}\underset{v\in S(l)}{\sum }\text{TC}(v)\; & \;\\ & =\underset{l\in \text{Anc}(e)}{\sum }w_{l}\left(\underset{v\in S}{\sum }\text{TC}\left(v\right)-\underset{x\in S(l)}{\sum }\text{TC}\left(x\right)\right)\;\\ & \;+\underset{l\in E}{\sum }w_{l}\underset{v\in S(l)}{\sum }\text{TC}(v)-\underset{l\in \text{Anc}(e)}{\sum }w_{l}\underset{v\in S(l)}{\sum }\text{TC}(v)\;.\; \end{array}$$

In the last expression, value $\underset{v\in S}{\sum }\text{TC}(v)$ can be computed in *O*(*n*) time, given that we have already computed TC(*v*) for every *v*∈*S*. Value $\underset{l\in E}{\sum }w_{l}\underset{v\in S(l)}{\sum }\text{TC}(v)$ and values $\underset{x\in S(l)}{\sum }\text{TC}(x)$ for any *l*∈*E* can be calculated with a bottom-up scan of  in a similar way as we computed *TC*_*s* *u* *b*_(*e*) for all *e*∈*E*. The remaining sums that involve edges in Anc(*e*) can be computed in linear time for every edge *e* with a similar mechanism as with SUM_3_(*e*) that we described earlier in this proof. For any edge *e*∈*E*, quantities PC(*e*) and PSQ(*e*) in Table 1 are equal to:

$$\begin{array}{ll} \text{PC}(e)= & \;\underset{u\in S}{\sum }\text{cost}(u,e)={\text{TC}}_{\text{sub}}(e)+\underset{l\in \text{Ind}(e)}{\sum }w_{l}\cdot s(l)\;\\ & +\underset{l\in \text{Anc}(e)}{\sum }w_{l}(s-s(l))\;,\; \end{array}$$

and:

$$\begin{array}{ll} \;\text{PSQ}(e) & =\underset{u\in S}{\sum }\text{cos}t^{2}(u,e)={\text{SQ}}_{\text{sub}}(e)\;\\ & \;+2\underset{l\in \text{Ind}(e)}{\sum }w_{l}\cdot {\text{TC}}_{\text{sub}}(l)+w_{l}^{2}\cdot s(l)\;\\ & \;+2\underset{l\in \text{Anc}(e)}{\sum }w_{l}\underset{k\in \text{Ind}(l)}{\sum }w_{k}\cdot s_{k}\;\\ & \;+2\underset{l\in \text{Anc}(e)}{\sum }\;w_{l}(s\;-\;s_{l})\;\;\left(\underset{\begin{array}{c} k\in \text{Anc}(e)\\ k\in \text{Off}(l) \end{array}}{\sum }w_{k}\right)\;+\;w_{l}^{2}(s\;-\;s(l)).\; \end{array}$$

From the two last expressions, and given the description that we provided for other similar quantities in Table 1, it easy to conclude that PC(*e*) can be evaluated for every edge *e* in *O*(*n*) time by scanning  a constant number of times. Having computed PC(*e*) for all edges *e*∈*E*, the quantity PSQ(*e*) can be computed in a similar manner.

Next we describe a divide-and-conquer approach for computing in *Θ*(*n*) time quantity (XI) in Table 1 for every *e*∈*E*. Before we start our description, we define one more quantity that will help us simplify the rest of this proof. For an edge *e*∈*E* and a tip *u*∈*S*(*e*) we define that TC_*e*_(*u*) is equal to:

$$\begin{array}{ll} & {\text{TC}}_{e}(u)=\underset{v\in S(e)\setminus \{u\}}{\sum }\text{cost}(u,v)\;.\; \end{array}$$

For any edge *e*∈*E* it is easy to show that:

(3) $$\begin{array}{ll} & \underset{u\in S(e)}{\sum }{\text{TC}}_{e}(u)=\underset{u\in S(e)}{\sum }\text{TC}(u)-\text{TC}(e)\; \end{array}$$

Therefore, according to (3) we can compute the sum $\underset{u\in S(e)}{\sum }{\text{TC}}_{e}(u)$ for all edges *e*∈*E* in linear time in total, given that we have already computed TC(*e*) for every *e*∈*E*, and TC(*u*) for every *u*∈*S*.

Next we continue our description for computing QD(*e*) using a divide-and-conquer approach. We start with the base case; for every edge tree *e* that is adjacent to a leaf node we have:

$$\begin{array}{ll} & \text{QD}(e)=\underset{u\in S(e)}{\sum }\;{\left(\underset{v\in S(e)\setminus \{u\}}{\sum }\text{cost}\left(u,,,v\right)\right)}^{2}=0.\; \end{array}$$

For any edge *e*∈*E* that is not adjacent to a leaf node, we can calculate QD(*e*) using the values of the respective quantities of the edges in Ch(*e*):

(4) $$\begin{array}{ll} \text{QD}(e)= & \;\underset{l\in \text{Ch}(e)}{\sum }\text{QD}(l)\;\\ & +2\underset{l\in \text{Ch}(e)}{\sum }\underset{u\in S(l)}{\sum }\underset{v\in S(e)\setminus S(l)}{\sum }\text{cost}(u,v)\cdot {\text{TC}}_{l}(u)\;\\ & +\underset{l\in \text{Ch}(e)}{\sum }\underset{u\in S(l)}{\sum }{\left(\underset{v\in S(e)\setminus S(l)}{\sum }\text{cost}\left(u,,,v\right)\right)}^{2}\;.\;\; \end{array}$$

The first sum in (4) can be computed in *Θ*(|Ch(*e*)|) time for each edge *e*, given that we have already computed the values QD(*l*) for every *l*∈Ch(*e*). We leave the description for calculating the second sum in (4) for the end of this proof. The third sum in this expression is equal to:

(5) $$\begin{array}{ll} & \underset{l\in \text{Ch}(e)}{\sum }\underset{u\in S(l)}{\sum }\;{\left(\underset{v\in S(e)\setminus S(l)}{\sum }\text{cost}\left(u,,,v\right)\right)}^{2}\;\\ & \;=\underset{l\in \text{Ch}(e)}{\sum }\underset{u\in S(l)}{\sum }\;{\left(\underset{v\in S(e)\setminus S(l)}{\sum }\text{cost}\left(u,,,l\right)+\text{cost}\left(v,,,l\right)\right)}^{2}\;\\ & \;=\underset{l\in \text{Ch}(e)}{\sum }\underset{u\in S(l)}{\sum }\underset{v\in S(e)\setminus S(l)}{\sum }\underset{x\in S(e)\setminus S(l)}{\sum }\text{cos}t^{2}(u,l)\;\\ & \;+\text{cost}(u,l)\cdot \text{cost}(v,l)+\text{cost}(u,l)\cdot \text{cost}(x,l)\;\\ & \;+\text{cost}(v,l)\cdot \text{cost}(x,l)\;.\; \end{array}$$

The first term of the sum in (5) can be expressed as:

(6) $$\begin{array}{ll} & \underset{l\in \text{Ch}(e)}{\sum }\underset{u\in S(l)}{\sum }\underset{v\in S(e)\setminus S(l)}{\sum }\underset{x\in S(e)\setminus S(l)}{\sum }\;\text{cos}t^{2}(u,l)\;\\ & \;=\underset{l\in \text{Ch}(e)}{\sum }\underset{u\in S(l)}{\sum }\;{\left(s(e)-s(l)\right)}^{2}\cdot \text{cos}t^{2}(u,l)\;\\ & \;=\underset{l\in \text{Ch}(e)}{\sum }{\left(s(e)-s(l)\right)}^{2}\cdot {\text{SQ}}_{\text{sub}}(l)\;,\; \end{array}$$

and can be computed in *Θ*(|Ch(*e*)|) time, given that we have already computed *SQ*_*s* *u* *b*_(*l*),∀*l*∈Ch(*e*).

The next two parts of the sum in (5) are equal to:

(7) $$\;\begin{array}{l} \underset{l\in \text{Ch}(e)}{\sum }\;\underset{u\in S(l)}{\sum }\;\underset{v\in S(e)\setminus S(l)}{\sum }\;\underset{x\in S(e)\setminus S(l)}{\sum }\;\text{cost}(u,l)\cdot \text{cost}(v,l)\\ \;+\text{cost}(u,l)\cdot \text{cost}(x,l)\\ \;=\underset{l\in \text{Ch}(e)}{\sum }\;\underset{u\in S(l)}{\sum }\;\underset{v\in S(e)\setminus S(l)}{\sum }2\cdot (s(e)-s(l))\cdot \text{cost}(u,l)\cdot \text{cost}(v,l)\\ \;=2\underset{l\in \text{Ch}(e)}{\sum }\;(s(e)-s(l))\;\underset{u\in S(l)}{\sum }\;\text{cost}(u,l)\;\left(w_{l}(s(e)-s(l))\right)\\ \;+\underset{k\in \text{Ch}(e)}{\sum }\;\left(w_{k}\cdot s\left(k\right)\right)-w_{l}\cdot s\left(l\right)\\ \;+\left(\left(-\underset{k\in \text{Ch}(e)}{\sum }\;{\text{TC}}_{\text{sub}}\left(k\right)\right)-{\text{TC}}_{\text{sub}}\left(l\right)\right)\\ \;=2\underset{l\in \text{Ch}(e)}{\sum }\;(s(e)-s(l))\cdot {\text{TC}}_{\text{sub}}\;\left(l\right)\cdot \left(w_{l}(s(e)-s(l))\right)\\ \;+\left(\underset{k\in \text{Ch}(e)}{\sum }w_{k}\cdot s\left(k\right)\right)-w_{l}\cdot s\left(l\right)+\underset{k\in \text{Ch}(e)}{\sum }\;{\text{TC}}_{\text{sub}}\left(k\right)\\ \;-\left({\text{TC}}_{\text{sub}}\left(l\right)\right)\;. \end{array}$$

The last expression can be computed in *Θ*(|Ch(*e*)|) time as well, if we have already computed the sum $\underset{k\in \text{Ch}(e)}{\sum }w_{k}\cdot s(k)$ and the quantity *TC*_*s* *u* *b*_(*e*) for every edge *e* in the tree. We can rewrite the remaining term in (5) as:

(8) $$\begin{array}{ll} & \;\underset{l\in \text{Ch}(e)}{\sum }\;\underset{u\in S(l)}{\sum }\;\underset{v\in S(e)\setminus S(l)}{\sum }\;\underset{x\in S(e)\setminus S(l)}{\sum }\;\text{cost}(v,l)\cdot \text{cost}(x,l)\;\\ & \;=\underset{l\in \text{Ch}(e)}{\sum }\;\underset{u\in S(l)}{\sum }\;{\left(\underset{v\in S(e)\setminus S(l)}{\sum }\;\text{cost}\left(v,,,l\right)\right)}^{2}\;\\ & \;=\underset{l\in \text{Ch}(e)}{\sum }\;s(l)\cdot {\left(\underset{v\in S(e)\setminus S(l)}{\sum }\;\text{cost}\left(v,,,l\right)\right)}^{2}\;\\ & \;=\underset{l\in \text{Ch}(e)}{\sum }\;s\left(l\right)\;\cdot \;\left(\;w_{l}(s(e)-s(l))+\;\;\underset{k\in \text{Ch}\left(e\right)}{\sum }\;\;w_{k}\cdot s\left(k\right)-w_{l}\;\cdot \;s\left(l\right)\right)\;\\ & +{\left(\underset{k\in \text{Ch}(e)}{\sum }\;{\text{TC}}_{\text{sub}}\;\left(k\right)-{\text{TC}}_{\text{sub}}\;\left(l\right)\right)}^{2}\;.\; \end{array}$$

The last expression can be computed in *Θ*(|Ch(*e*)|) time in a similar way as the previous terms of the sum in (5).

We left for the end the description of the calculation of the second sum in (4). We can express this sum as follows:

(9) $$\begin{array}{ll} & \;\underset{l\in \text{Ch}(e)}{\sum }\;\underset{u\in S(l)}{\sum }\;\underset{v\in S(e)\setminus S(l)}{\sum }\;\text{cost}(u,v)\cdot {\text{TC}}_{l}(u)\;\\ & \;=\underset{l\in \text{Ch}(e)}{\sum }\;\underset{u\in S(l)}{\sum }\;\underset{v\in S(e)\setminus S(l)}{\sum }\;\left(\text{cost}(u,l)+\text{cost}(v,l)\right)\cdot {\text{TC}}_{l}(u)\;\\ & \;=\underset{l\in \text{Ch}(e)}{\sum }\;\underset{u\in S(l)}{\sum }\;\underset{v\in S(e)\setminus S(l)}{\sum }\;\text{cost}(u,l)\cdot {\text{TC}}_{l}(u)\;\\ & +\underset{l\in \text{Ch}(e)}{\sum }\;\underset{u\in S(l)}{\sum }\;\underset{v\in S(e)\setminus S(l)}{\sum }\;\text{cost}(v,l)\cdot {\text{TC}}_{l}(u)\;\\ & \;=\underset{l\in \text{Ch}(e)}{\sum }\;\underset{u\in S(l)}{\sum }\;(s(e)-s(l))\cdot \text{cost}(u,l)\cdot {\text{TC}}_{l}(u)\;\\ & +\underset{l\in \text{Ch}(e)}{\sum }\;\underset{u\in S(l)}{\sum }\;\underset{v\in S(e)\setminus S(l)}{\sum }\;\text{cost}(v,l)\cdot {\text{TC}}_{l}(u)\;.\; \end{array}$$

We start with the second sum in (9). For this sum we get:

$$\begin{array}{ll} & \;\underset{l\in \text{Ch}(e)}{\sum }\;\underset{u\in S(l)}{\sum }\;\underset{v\in S(e)\setminus S(l)}{\sum }\;\text{cost}(v,l)\cdot {\text{TC}}_{l}(u)\;\\ & \;=\underset{l\in \text{Ch}\left(e\right)}{\sum }\;\underset{u\in S\left(l\right)}{\sum }\;\left(w_{l}\left(s,\left(e\right),-,s,\left(l\right)\right)+\;\left(\underset{k\in \text{Ch}\left(e\right)}{\sum }w_{k}\cdot s\left(k\right)\right)\right)\;\\ & -w_{l}\cdot s\left(l\right)+\;\;\left(\left(\underset{k\in \text{Ch}\left(e\right)}{\sum }\;{\text{TC}}_{\text{sub}}\left(k\right)\right)\;-\;{\text{TC}}_{\text{sub}}\left(l\right)\right)\;\cdot {\text{TC}}_{l}\left(u\right).\; \end{array}$$

Because of (3), the last expression can be written as:

$$\begin{array}{ll} & \;\underset{l\in \text{Ch}\left(e\right)}{\sum }\;\underset{u\in S\left(l\right)}{\sum }\;\left(w_{l}\left(s\right)\left(e\right)-s\left(\left(l\right)\right)+\;\underset{k\in \text{Ch}(e)}{\sum }\;\left(w_{k}\cdot s\left(k\right)\right)-w_{l}\cdot s\left(l\right)\right)\;\\ & +\left(\left(\underset{k\in \text{Ch}(e)}{\sum }\;{\text{TC}}_{\text{sub}}\left(k\right)\right)-{\text{TC}}_{\text{sub}}\left(l\right)\right)\cdot {\text{TC}}_{l}(u)\;\\ & \;=\underset{l\in \text{Ch}(e)}{\sum }\;\left(w_{l}\left(s\right)\left(e\right)-s\left(\left(l\right)\right)+\left(\underset{k\in \text{Ch}(e)}{\sum }\;w_{k}\cdot s\left(k\right)\right)-w_{l}\cdot s\left(l\right)\right)\;\\ & +\left(\left(\underset{k\in \text{Ch}(e)}{\sum }\;{\text{TC}}_{\text{sub}}\left(k\right)\right)\;-\;{\text{TC}}_{\text{sub}}\left(l\right)\right)\;\;\;\left(\;\underset{u\in S(e)}{\sum }\;\text{TC}\left(u\right)\;-\;\text{TC}\left(e\right)\;\right)\;,\; \end{array}$$

which takes *Θ*(|Ch(*e*)|) time to be computed for each edge *e*.

To compute the first sum in (9) efficiently, we need to precompute for every edge *l*∈*E* the following quantity:

$$\underset{u\in S(e)}{\sum }\text{cost}\left(u,,,e\right)\cdot {\text{TC}}_{e}\left(u\right)\;.$$

To do this, we follow again a divide-and-conquer approach. We get the base case for this computation for the edges of  that are adjacent to tips. For any such edge *e* we have:

$$\underset{u\in S(e)}{\sum }\text{cost}\left(u,,,e\right)\cdot {\text{TC}}_{e}\left(u\right)=0\;.$$

For any other edge *e*∈*E* we can compute this quantity based on the respective quantities of the edges in Ch(*e*). In particular, we have that:

(10) $$\;\begin{array}{l} \underset{u\in S(e)}{\sum }\;\text{cost}(u,e)\cdot {\text{TC}}_{e}(u)=\underset{l\in \text{Ch}(e)}{\sum }\;\underset{u\in S(l)}{\sum }\;\text{cost}(u,l)\cdot {\text{TC}}_{l}(u)\\ \;\;+\underset{l\in \text{Ch}(e)}{\sum }\;w_{l}\underset{u\in S(l)}{\sum }\;{\text{TC}}_{l}(u)+\underset{l\in \text{Ch}(e)}{\sum }\\ \;\;\underset{u\in S(l)}{\sum }\;\underset{v\in S(e)\setminus S(l)}{\sum }\;\text{cost}(u,e)\cdot \text{cost}(u,v)\\ \;=\;\underset{l\in \text{Ch}(e)}{\sum }\;\underset{u\in S(l)}{\sum }\;\text{cost}(u,l)\;\cdot \;{\text{TC}}_{l}(u)\;+\;\underset{l\in \text{Ch}(e)}{\sum }\\ \;\;\times \;w_{l}\;\left(\underset{u\in S(l)}{\sum }\text{TC}\left(u\right)\;-\;\text{TC}\left(l\right)\right)\;+\;\underset{l\in \text{Ch}(e)}{\sum }\\ \;\;\underset{u\in S(l)}{\sum }\;\underset{v\in S(e)\setminus S(l)}{\sum }\;\text{cost}(u,e)\cdot \text{cost}(u,v)\;. \end{array}$$

The first two sums in the last expression can be computed in *Θ*(|Ch(*e*)|) time, given that we have computed already for every *l*∈Ch(*e*) the quantity TC(*l*) and the sum $\underset{u\in S(l)}{\sum }\text{TC}(u)$ (can be done with a single bottom-up scan of the tree). The last sum in (10) can be expressed as:

(11) $$\;\begin{array}{l} \;\underset{l\in \text{Ch}(e)}{\sum }\;\underset{u\in S(l)}{\sum }\;\underset{v\in S(e)\setminus S(l)}{\sum }\;\text{cost}(u,e)\cdot \text{cost}(u,v)\\ \;=\underset{l\in \text{Ch}(e)}{\sum }\;w_{l}\;\underset{u\in S(l)}{\sum }\;\underset{v\in S(e)\setminus S(l)}{\sum }\;\text{cost}(u,v)\\ \;+\;\underset{l\in \text{Ch}(e)}{\sum }\;\underset{u\in S(l)}{\sum }\;\underset{v\in S(e)\setminus S(l)}{\sum }\;\text{cost}(u,l)\cdot \text{cost}(u,v)\\ \;=\underset{l\in \text{Ch}(e)}{\sum }\;w_{l}\underset{u\in S(l)}{\sum }\;\underset{v\in S(e)\setminus S(l)}{\sum }\;\text{cost}(u,v)\\ \;+\;\underset{l\in \text{Ch}(e)}{\sum }\;\underset{u\in S(l)}{\sum }\;\underset{v\in S(e)\setminus S(l)}{\sum }\;\text{cos}t^{2}(u,l)\\ \;+\underset{l\in \text{Ch}(e)}{\sum }\;\underset{u\in S(l)}{\sum }\;\underset{v\in S(e)\setminus S(l)}{\sum }\;\text{cost}(u,l)\cdot \text{cost}(v,l)\;. \end{array}$$

The two last sums in (11) are identical with the quantities that we analysed in (6) and in (7). Finally, the first sum in (11) is equal to:

(12) $$\;\begin{array}{l} \underset{l\in \text{Ch}(e)}{\sum }\;w_{l}\;\underset{u\in S(l)}{\sum }\;\underset{v\in S(e)\setminus S(l)}{\sum }\;\text{cost}(u,v)\\ \;=\underset{l\in \text{Ch}(e)}{\sum }\;w_{l}(s(e)-s(l))\cdot {\text{TC}}_{\text{sub}}(l)\\ \;+\underset{l\in \text{Ch}(e)}{\sum }\;w_{l}^{2}\cdot s(l)\cdot (s(e)-s(l))\\ \;+\underset{l\in \text{Ch}(e)}{\sum }\;w_{l}\left(s\left(l\right)\;\left(\underset{k\in \text{Ch}(e)}{\sum }\;s\left(k\right)\cdot w_{k}\right)-s^{2}\left(l\right)\cdot w_{l}\right)\\ \;+\underset{l\in \text{Ch}(e)}{\sum }\;w_{l}\cdot s(l)\left(\left(\underset{k\in \text{Ch}(e)}{\sum }{\text{TC}}_{\text{sub}}\left(k\right)\right)\;-\;{\text{TC}}_{\text{sub}}\left(l\right)\right), \end{array}$$

which can also be computed in *Θ*(|Ch(*e*)|) time.

All the sums that we analysed from (4) up to (12) can be computed in *Θ*(|Ch(*e*)|) time for every edge *e* in the tree. From this we conclude that for every edge *e*∈*E* we can evaluate QD(*e*) in (4) in *Θ*(|Ch(*e*)|) time from the respective values of the edges in Ch(*e*). Since $\underset{e\in E}{\sum }|\text{Ch}(e)|=\Theta (|E|)$, we prove that we can compute QD(*e*) for all the edges in  in *Θ*(*n*).

## Computing the skewness of the MPD

In the previous section we defined the problem of computing the skewness of the MPD for a given phylogenetic tree . Given a positive integer *r*≤*s*, we showed that to solve this problem efficiently it remains to find an efficient algorithm for computing $E_{R\in \text{Sub}(S,r)}[{\text{MPD}}^{3}(T,R)]$; this is the mean value of the cube of the MPD among all possible subsets of tips in  that consist of exactly *r* elements. To compute this efficiently, we introduced in Table 1 eleven different quantities which we want to use in order to express this mean value. In Lemma 1 we proved that these quantities can be computed in *O*(*n*) time, where *n* is the size of .

Next we prove how we can calculate the value for the mean of the cube of the MPD based on the quantities in Table 1. In particular, in the proof of the following lemma we show how the value $E_{R\in \text{Sub}(S,r)}[{\text{MPD}}^{3}(T,R)]$ can be written analytically as an expression that contains the quantities in Table 1. This expression can then be straightforwardly evaluated in *O*(*n*) time, given that we have already computed the aforementioned quantities. Because the full form of this expression is very long (it consists of a large number of terms), we have chosen not to include it in the definition of the following lemma. We chose to do so because we considered that including the entire expression would not make this work more readable. In any case, the full expression can be easily infered from the proof of the lemma.

### 

**Lemma 2. ***For any given natural r ≤ s, we can compute *$E_{R\in \text{Sub}(S,r)}[{\text{MPD}}^{3}(T,R)]$*in Θ (n) time.*

### 

*Proof.* The expectation of the cube of the MPD is equal to:

$$\begin{array}{ll} & \;E_{R\in \text{Sub}(S,r)}[{\text{MPD}}^{3}(T,R)]\;\\ & \;=\frac{8}{r^{3}{\left(r-1\right)}^{3}}\cdot E_{R\in \text{Sub}(S,r)}\;\left[\underset{\left\{u,v\}\in \Delta (R\right)}{\sum }\;\underset{\left\{x,y\}\in \Delta (R\right)}{\sum }\;\underset{\left\{c,d\}\in \Delta (R\right)}{\sum }\right)\;\\ & \;\;\;\;\;\left(\text{cost}(u,v)\cdot \text{cost}(x,y)\cdot \text{cost}(c,d)\right]\;.\; \end{array}$$

From the last expression we get:

(13) $$\begin{array}{ll} & E_{R\in \text{Sub}(S,r)}\left[\underset{\left\{u,v\}\in \Delta (R\right)}{\sum }\;\underset{\left\{x,y\}\in \Delta (R\right)}{\sum }\;\underset{\left\{c,d\}\in \Delta (R\right)}{\sum }\right)\;\\ & \left(\;\;\;\;\;\text{cost}(u,v)\cdot \text{cost}(x,y)\cdot \text{cost}(c,d)\right]\;\\ & =\underset{\left\{u,v\}\in \Delta (S\right)}{\sum }\;\underset{\left\{x,y\}\in \Delta (S\right)}{\sum }\;\underset{\left\{c,d\}\in \Delta (S\right)}{\sum }\;\text{cost}(u,v)\cdot \text{cost}(x,y)\;\\ & \;\cdot \text{cost}(c,d)\cdot E_{R\in \text{Sub}(S,r)}[{\text{AP}}_{R}(u,v,x,y,c,d)]\;,\; \end{array}$$

where *AP*_*R*_(*u*,*v*,*x*,*y*,*c*,*d*) is a random variable whose value is equal to one in the case that *u*,*v*,*x*,*y*,*c*,*d*∈*R*, otherwise it is equal to zero. For any six tips *u*,*v*,*x*,*y*,*c*,*d*∈*S*, which may not be all of them distinct, we use *θ*(*u*,*v*,*x*,*y*,*c*,*d*) to denote the number of distinct elements among these tips. Let *t* be an integer, and let (*t*)_*k*_ denote the *k*-th falling factorial power of *t*, which means that (*t*)_*k*_=*t*(*t*-1)…(*t*-*k*+1). For the expectation of the random variables that appear in the last expression it holds that:

(14) $$\begin{array}{l} E_{R\in \text{Sub}(S,r)}\left[AP_{R}(u,v,x,y,c,d)\right]=\frac{{\left(r\right)}_{\theta (u,v,x,y,c,d)}}{{\left(s\right)}_{\theta (u,v,x,y,c,d)}} \end{array}$$

Notice that in (14) we have 2≤*θ*(*u*,*v*,*x*,*y*,*c*,*d*)≤6. The value of the function *θ*(·) cannot be smaller than two in the above case because we have that *u*≠*v*, *x*≠*y*, and *c*≠*d*. Thus, we can rewrite (13) as:

(15) $$\begin{array}{ll} & \underset{\left\{u,v\}\in \Delta (S\right)}{\sum }\;\underset{\left\{x,y\}\in \Delta (S\right)}{\sum }\;\underset{\left\{c,d\}\in \Delta (S\right)}{\sum }\;\frac{{\left(r\right)}_{\theta (u,v,x,y,c,d)}}{{\left(s\right)}_{\theta (u,v,x,y,c,d)}}\cdot \text{cost}(u,v)\;\\ & \;\cdot \text{cost}(x,y)\cdot \text{cost}(c,d)\; \end{array}$$

Hence, our goal now is to compute a sum whose elements are the product of costs of triples of paths. Recall that for each of these paths, the end-nodes of the path are a pair of distinct tips in the tree. Although the end-nodes of each path are distinct, in a given triple the paths may share one or more end-nodes with each other. Therefore, the distinct tips in any triple of paths may vary from two up to six tips. Indeed, in (15) we get a sum where the triples of paths in the sum are partitioned in five groups; a triple of paths is assigned to a group depending on the number of distinct tips in this triple. In (15) the sum for each group of triples is multiplied by the same factor (*r*)_*θ*(*u*,*v*,*x*,*y*,*c*,*d*)_/(*s*)_*θ*(*u*,*v*,*x*,*y*,*c*,*d*)_, hence we have to calculate the sum for each group of triples separately.

**Figure 1:**
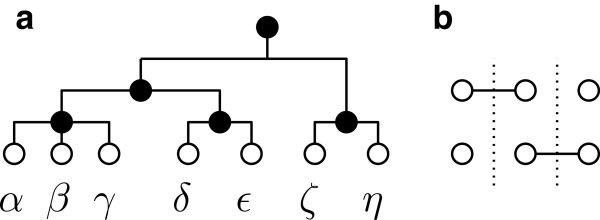
**Representing triples of paths as graphs. (a)** A phylogenetic tree  and **(b)** an example of the tripartite graph induced by the triplet of its tip pairs {*α*,*γ*},{*δ*,*γ*},{*ε*,*δ*}, where {*α*,*γ*,*δ*,*ε*}⊂*S*. The dashed lines in the graph distinguish the partite subsets of vertices; the vertices of each partite subset correspond to tips of the same pair.

However, when we try to calculate the sum for each of these groups of triples we see that this calculation is more involved; some of these groups of triples are divided into smaller subgroups, depending on which end-nodes of the paths in each triple are the same. To explain this better, we can represent a triple of paths schematically as a graph; let {*u*,*v*},{*x*,*y*},{*c*,*d*}∈*Δ*(*S*) be three pairs of tips in . As mentioned already, the tips within each pair are distinct, but tips between different pairs can be the same.We represent the similarity between tips of these three pairs as a graph of six vertices. Each vertex in the graph corresponds to a tip of these three pairs. Also, there exists an edge in this graph between two vertices if the corresponding tips are the same. Thus, this graph is tripartite; no vertices that correspond to tips of the same pair can be connected to each other with an edge. Hence, we have a tripartite graph where each partite set of vertices consists of two vertices–see Figure 1 for an example. 

**Figure 2:**
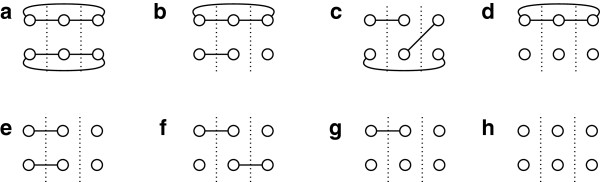
**Isomorphism classes.** The eight isomorphism classes of a tripartite graph of 3×2 vertices that represent schematically the eight possible cases of similarities between tips that we can have when we consider three paths between pairs of tips in a tree .

For any triple of pairs of tips {*u*,*v*}, {*x*,*y*}, {*c*,*d*}∈*Δ*(*S*) we denote the tripartite graph that corresponds to this triple by *G*[ *u*,*v*,*x*,*y*,*c*,*d*]. We call this graph the *similarity* graph of this triple. Based on the way that similarities may occur between tips in a triple of paths, we can partition the five groups of triples in (15) into smaller subgroups. Each of these subgroups contains triples whose similarity graphs are isomorphic. For a tripartite graph that consists of three partite sets of two vertices each, there can be eight different isomorphism classes. Therefore, the five groups of triples in (15) are partitioned into eight subgroups. Figure 2 illustrates the eight isomorphism classes that exist for the specific kind of tripartite graphs that we consider. Since we refer to isomorphism classes, each of the graphs in Figure 2 represents the combinatorial structure of the similarities between three pairs of tips, and it does not correspond to a particular planar embedding, or ordering of the tips. 

Let *X* be any isomorphism class that is illustrated in Figure 2. We denote the set of all triples of pairs in *Δ*(*S*) whose similarity graphs belong to this class by ${ℬ}_{X}$. More formally, the set ${ℬ}_{X}$ can be defined as follows :

$$\begin{array}{l} {ℬ}_{X}=\left\{\left\{\{u,v\},\{x,y\},\{c,d\}\}:\{u,v\},\{x,y\},\{c,d\}\in \Delta (S\right)\right)\\ \left(\text{and}\;G[u,v,x,y,c,d]\;\text{belongs to class}\;X\;\text{in Figure 2}\right\}\;. \end{array}$$

We introduce also the following quantity:

$$\;\text{TRS}(X)=\underset{\{\{u,v\},\{x,y\},\{c,d\}\}\in {ℬ}_{X}}{\sum }\text{cost}\left(u,,,v\right)\cdot \text{cost}\left(x,,,y\right)\cdot \text{cost}\left(c,,,d\right)\;.$$

Hence, we can rewrite (15) as follows:

(16) $$\begin{array}{l} \;\frac{{\left(r\right)}_{2}}{{\left(s\right)}_{2}}\cdot \text{TRS}(A)+3\cdot \frac{{\left(r\right)}_{3}}{{\left(s\right)}_{3}}\cdot \text{TRS}(B)+6\cdot \frac{{\left(r\right)}_{3}}{{\left(s\right)}_{3}}\cdot \text{TRS}(C)\\ \;+6\cdot \frac{{\left(r\right)}_{4}}{{\left(s\right)}_{4}}\cdot \text{TRS}(D)+3\cdot \frac{{\left(r\right)}_{4}}{{\left(s\right)}_{4}}\cdot \text{TRS}(E)+6\cdot \frac{{\left(r\right)}_{4}}{{\left(s\right)}_{4}}\cdot \text{TRS}(F)\\ \;+6\cdot \frac{{\left(r\right)}_{5}}{{\left(s\right)}_{5}}\cdot \text{TRS}(G)+6\cdot \frac{{\left(r\right)}_{6}}{{\left(s\right)}_{6}}\cdot \text{TRS}(H) \end{array}$$

Notice that some of the terms $\frac{{\left(r\right)}_{i}}{{\left(s\right)}_{i}}\cdot \text{TRS}(X)$ in (16) are multiplied with an extra constant factor. This happens for the following reason; the sum in TRS(*X*) counts each triple once for every different combination of three pairs of tips. However, in the triple sum in (15) some triples appear more than once. For example, every triple that belongs in class *B* appears three times in (15), hence there is an extra factor three in front of TRS(*B*) in (16).

To compute efficiently $E_{R\in \text{Sub}(S,r)}[{\text{MPD}}^{3}(T,R)]$, it remains to compute efficiently each value TRS(*X*) for every isomorphism class *X* that is presented in Figure 2. Next we show in detail how we can do that by expressing each quantity TRS(*X*) as a function of the quantities that appear in Table 1.

For the triples that correspond to the isomorphism class *A* we have:

$$\begin{array}{lll} & \text{TRS}(A)=\underset{\left\{u,v\}\in \Delta (S\right)}{\sum }\text{cos}t^{3}(u,v)=\text{CB}(T)\;.\; & \; \end{array}$$

For TRS(*B*) we get:

$$\begin{array}{ll} \;\text{TRS}(B) & =\underset{\left\{u,v\}\in \Delta (S\right)}{\sum }\text{cos}t^{2}\left(u,,,v\right)\left(\underset{x\in S\setminus \{u\}}{\sum }\text{cost}\left(u,,,x\right)\right)\;\\ & \;+\left(\underset{y\in S\setminus \{v\}}{\sum }\text{cost}\left(v,,,y\right)-2\cdot \text{cost}\left(u,,,v\right)\right)\;\\ & =\;\underset{\left\{u,v\}\in \Delta (S\right)}{\sum }\;\text{cos}t^{2}(u,v)\;\left(\text{TC}(u)\;+\;\text{TC}(v)\;-\;2\;\cdot \;\text{cost}(u,\;v)\;\right)\;\\ & =\underset{u\in S}{\sum }\text{SQ}(u)\cdot \text{TC}(u)-2\cdot \text{CB}(T)\;.\; \end{array}$$

The quantity TRS(*C*) is equal to:

(17) $$\begin{array}{ll} & \;\frac{1}{6}\underset{u\in S}{\sum }\;\underset{v\in S\setminus \{u\}}{\sum }\;\text{cost}(u,v)\underset{x\in S\setminus \{u,v\}}{\sum }\;\text{cost}(u,x)\cdot \text{cost}(x,v)\;\\ & \;=\underset{e\in E}{\sum }\;w_{e}\underset{u\in S(e)}{\sum }\;\underset{v\in S-S(e)}{\sum }\;\underset{x\in S\setminus \{u,v\}}{\sum }\;\text{cost}(u,x)\cdot \text{cost}(x,v)\;.\; \end{array}$$

For any *e*∈*E* we have that:

(18) $$\begin{array}{ll} & \;\underset{u\in S(e)}{\sum }\;\underset{v\in S-S(e)}{\sum }\;\underset{x\in S\setminus \{u,v\}}{\sum }\;\text{cost}(u,x)\cdot \text{cost}(x,v)\;\\ & =\underset{u\in S(e)}{\sum }\;\underset{v\in S\setminus \{u\}}{\sum }\;\underset{x\in S\setminus \{u,v\}}{\sum }\;\text{cost}(u,x)\cdot \text{cost}(x,v)\;\\ & \;-2\underset{\left\{u,v\}\in \Delta (S(e)\right)}{\sum }\;\underset{x\in S\setminus \{u,v\}}{\sum }\;\text{cost}(u,x)\cdot \text{cost}(x,v)\;.\; \end{array}$$

The first of the two sums in (18) can be written as:

(19) $$\begin{array}{ll} & \underset{u\in S(e)}{\sum }\;\underset{v\in S\setminus \{u\}}{\sum }\;\underset{x\in S\setminus \{u,v\}}{\sum }\;\text{cost}(u,x)\cdot \text{cost}(x,v)\;\\ & =\underset{u\in S(e)}{\sum }\;\underset{v\in S\setminus \{u\}}{\sum }\;\underset{x\in S\setminus \{u,v\}}{\sum }\;\text{cost}(u,v)\cdot \text{cost}(x,v)\;\\ & =\underset{u\in S(e)}{\sum }\;\underset{v\in S\setminus \{u\}}{\sum }\;(\text{cost}(u,v)\cdot \text{TC}(v)-\text{cos}t^{2}(u,v))\;\\ & =\underset{u\in S(e)}{\sum }\;\text{SM}(u)-\text{SQ}(u)\;.\; \end{array}$$

According to Lemma 2, we can compute SM(*u*) and SQ(*u*) for all tips *u*∈*S* in linear time with respect to the size of . Given these values, we can compute $\underset{u\in S(e)}{\sum }\text{SM}(u)-\text{SQ}(u)$ for every edge *e*∈*E* in  with a single bottom-up scan of the tree. For any edge *e* in *E*, the second sum in (18) is equal to:

(20) $$\begin{array}{ll} & \;\underset{\left\{u,v\}\in \Delta (S(e)\right)}{\sum }\;\underset{x\in S\setminus \{u,v\}}{\sum }\;\text{cost}(u,x)\cdot \text{cost}(x,v)\;\\ & =\underset{\left\{u,v\}\in \Delta (S(e)\right)}{\sum }\;\underset{x\in S(e)\setminus \{u,v\}}{\sum }\text{cost}(u,x)\cdot \text{cost}(x,v)\;\\ & \;+\underset{\left\{u,v\}\in \Delta (S(e)\right)}{\sum }\;\underset{x\in S\setminus S(e)}{\sum }\;\text{cost}(u,x)\cdot \text{cost}(x,v)\;.\; \end{array}$$

We can express the first sum in (20) as:

(21) $$\begin{array}{ll} & \;\underset{\left\{u,v\}\in \Delta (S(e)\right)}{\sum }\;\underset{x\in S(e)\setminus \{u,v\}}{\sum }\;\text{cost}(u,x)\cdot \text{cost}(x,v)\;\\ & =\frac{1}{2}\underset{u\in S(e)}{\sum }\;{\left(\underset{v\in S(e)\setminus \{u\}}{\sum }\;\text{cost}\left(u,,,v\right)\right)}^{2}\;\\ & \;-\frac{1}{2}\underset{u\in S(e)}{\sum }\;\underset{v\in S(e)\setminus \{u\}}{\sum }\;\text{cos}t^{2}(u,v)\;\\ & =\frac{1}{2}\text{QD}(e)-\frac{1}{2}\underset{u\in S(e)}{\sum }\;\underset{v\in S(e)\setminus \{u\}}{\sum }\;\text{cos}t^{2}(u,v)\;.\; \end{array}$$

The last sum in (21) is equal to:

(22) $$\begin{array}{ll} & \underset{u\in S(e)}{\sum }\underset{v\in S(e)\setminus \{u\}}{\sum }\text{cos}t^{2}(u,v)=\left(\underset{u\in S(e)}{\sum }\text{SQ}\left(u\right)\right)-\text{SQ}\left(e\right)\;.\; \end{array}$$

The value of the sum $\underset{u\in S(e)}{\sum }\text{SQ}(u)$ can be computed for every edge *e* in *Θ*(*n*) time in total as follows; for every tip *u*∈*S* we store SQ(*u*) together with this tip, and then scan bottom-up the tree adding those values that are in the subtree of each edge. For the remaining part of (20) we get:

(23) $$\begin{array}{ll} & \;\underset{\left\{u,v\}\in \Delta (S(e)\right)}{\sum }\;\underset{x\in S\setminus S(e)}{\sum }\;\text{cost}(u,x)\cdot \text{cost}(x,v)\;\\ & =\underset{\left\{u,v\}\in \Delta (S(e)\right)}{\sum }\;\underset{x\in S\setminus S(e)}{\sum }\;\left(\text{cost}(u,e)+\text{cost}(x,e)\right)\;\\ & \;\times \left(\text{cost}(v,e)+\text{cost}(x,e)\right)\;\\ & =\underset{\left\{u,v\}\in \Delta (S(e)\right)}{\sum }\;\underset{x\in S\setminus S(e)}{\sum }\;\text{cost}(u,e)\cdot \text{cost}(v,e)\;\\ & \;+\;\underset{\left\{u,v\}\in \Delta (S(e)\right)}{\sum }\;\underset{x\in S\setminus S(e)}{\sum }\;\text{cost}(x,e)\;\cdot \;(\text{cost}(u,e)\;+\;\text{cost}(v,e))\;\\ & \;+\underset{\left\{u,v\}\in \Delta (S(e)\right)}{\sum }\;\underset{x\in S\setminus S(e)}{\sum }\;\text{cos}t^{2}(x,e)\;.\; \end{array}$$

The first sum in (23) is equal to:

(24) $$\begin{array}{ll} & \underset{\left\{u,v\}\in \Delta (S(e)\right)}{\sum }\underset{x\in S\setminus S(e)}{\sum }\text{cost}(u,e)\cdot \text{cost}(v,e)\;\\ & =\frac{1}{2}\cdot (s-s(e))\left({TC_{\text{sub}}}^{2}(e)-{\text{SQ}}_{\text{sub}}(e)\right)\;.\; \end{array}$$

For the second sum in (23) we have:

(25) $$\begin{array}{lll} & \;\underset{\left\{u,v\}\in \Delta (S(e)\right)}{\sum }\underset{x\in S\setminus S(e)}{\sum }\text{cost}(x,e)\cdot (\text{cost}(u,e)+\text{cost}(v,e))\;\\ & =(s(e)-1)\cdot {\text{TC}}_{\text{sub}}(e)\underset{x\in S\setminus S(e)}{\sum }\text{cost}(x,e)\; & \;\\ & =(s(e)-1)\cdot {\text{TC}}_{\text{sub}}(e)\cdot \left(\text{PC}(e)-{\text{TC}}_{\text{sub}}(e)\right)\;.\; \end{array}$$

The last sum in (23) can be written as:

(26) $$\begin{array}{ll} & \;\underset{\left\{u,v\}\in \Delta (S(e)\right)}{\sum }\underset{x\in S\setminus S(e)}{\sum }\text{cos}t^{2}(x,e)\;\\ & =\frac{s(e)(s(e)-1)}{2}\left(\text{PSQ}(e)-{\text{SQ}}_{\text{sub}}(e)\right)\;.\; \end{array}$$

Combining the analyses that we did from (17) up to (26) we get:

$$\begin{array}{ll} \;\text{TRS}(C)= & \;\underset{e\in E}{\sum }w_{e}\left(\underset{u\in S(e)}{\sum }\text{SM}\left(u\right)-\text{QD}\left(e\right)-\text{SQ}\left(e\right)\right)\;\\ & -(s-s(e))\left({{\text{TC}}_{\text{sub}}}^{2}(e)-{\text{SQ}}_{\text{sub}}(e)\right)\;\\ & -2(s(e)-1)\cdot {\text{TC}}_{\text{sub}}(e)\cdot \left(\text{PC}(e)-{\text{TC}}_{\text{sub}}(e)\right)\;\\ & \left(-\;s(e)(s(e)-1)\cdot \left(\text{PSQ}(e)-{\text{SQ}}_{\text{sub}}(e)\right)\right)\;.\; \end{array}$$

The value of TRS(*D*) can be expressed as:

$$\begin{array}{ll} & \;\underset{u\in S}{\sum }\underset{\begin{array}{c} v,x,y\in S\setminus \{u\}\\ v,x,y\text{are distinct} \end{array}}{\sum }\text{cost}(u,v)\cdot \text{cost}(u,x)\cdot \text{cost}(u,y)\;\\ & \;=\frac{1}{6}\left(\underset{u\in S}{\sum }{\text{TC}}^{3}\left(u\right)-2\cdot \text{TRS}\left(A\right)-3\cdot \text{TRS}\left(B\right)\right)\;\\ & \;=\frac{1}{6}\cdot \underset{u\in S}{\sum }{\text{TC}}^{3}(u)+\frac{2}{3}\cdot \text{CB}(T)-\frac{1}{2}\cdot \underset{u\in S}{\sum }\text{SQ}(u)\cdot \text{TC}(u)\;.\; \end{array}$$

For TRS(*E*) we get:

$$\begin{array}{ll} & \;\underset{\left\{u,v\}\in \Delta (S\right)}{\sum }\underset{\left\{x,y\}\in \Delta (S\setminus \{u,v\}\right)}{\sum }\text{cos}t^{2}(u,v)\cdot \text{cost}(x,y)\;\\ & \;=\underset{\left\{u,v\}\in \Delta (S\right)}{\sum }\text{cos}t^{2}(u,v)(\text{TC}(T)-\text{TC}(u)-\text{TC}(v)+\text{cost}(u,v))\;\\ & \;=\text{TC}(T)\underset{e\in E}{\sum }w_{e}\cdot \text{TC}(e)-\underset{u\in S}{\sum }\left(\text{SQ}(u)\cdot \text{TC}(u)\right)+\text{CB}(T)\;.\; \end{array}$$

We can rewrite TRS(*F*) as follows:

$$\begin{array}{ll} & \;\underset{\left\{u,v\}\in \Delta (S\right)}{\sum }\text{cost}(u,v)\;\\ & \;\left(\text{TC}\left(u\right)\cdot \text{TC}\left(v\right)-\text{cos}t^{2}\left(u,,,v,)\right)\underset{x\in S\setminus \{u,v\}}{\sum }\text{cost}\left(u,,,x\right)\cdot \text{cost}\left(x,,,v\right)\right)\;\\ & \;=\underset{\left\{u,v\}\in \Delta (S\right)}{\sum }\text{cost}(u,v)\cdot \text{TC}(u)\cdot \text{TC}(v)-\text{CB}(T)-3\cdot \text{TRS}(C)\;\\ & \;=\underset{e\in E}{\sum }w_{e}\cdot \text{Mult}(e)-\text{CB}(T)-3\cdot \text{TRS}(C)\;.\; \end{array}$$

For the value of TRS(*G*) we have:

(27) $$\begin{array}{ll} \;\text{TRS}(G) & =\frac{1}{2}\underset{\left\{u,v\}\in \Delta (S\right)}{\sum }\text{cost}(u,v)\underset{x\in S\setminus \{u,v\}}{\sum }\left(\text{cost}(u,x)\right)\;\\ & \;+\left(\text{cost}(v,x)\right)\left(\text{TC}(T)-\text{TC}(u)-\text{TC}(v)\right)\;\\ & \;-\left(\text{TC}(x)+\text{cost}(u,v)+\text{cost}(u,x)+\text{cost}(v,x)\right).\; \end{array}$$

We now break the sum in (27) into five pieces and express each piece of this sum in terms of the quantities in Table 1. The first piece of the sum is equal to:

$$\begin{array}{ll} & \;\frac{1}{2}\underset{\left\{u,v\}\in \Delta (S\right)}{\sum }\text{cost}(u,v)\underset{x\in S\setminus \{u,v\}}{\sum }\;\left(\text{cost}(u,x)\;+\;\text{cost}(v,x)\right)\;\cdot \;\text{TC}(T)\;\\ & \;=\frac{1}{2}\cdot \text{TC}(T)\left(\underset{u\in S}{\sum }{\text{TC}}^{2}\left(u,)\right)2\cdot \underset{\left\{u,v\}\in \Delta (S\right)}{\sum }\text{cos}t^{2}\left(u,,,v\right)\right)\;\\ & \;=\frac{1}{2}\cdot \text{TC}(T)\left(\underset{u\in S}{\sum }{\text{TC}}^{2}\left(u,)\right)2\cdot \underset{e\in E}{\sum }w_{e}\cdot \text{TC}\left(e\right)\right)\;.\; \end{array}$$

The second piece that we take from the sum in (27) can be expressed as:

(28) $$\;\begin{array}{l} \;-\frac{1}{2}\underset{\left\{u,v\}\in \Delta (S\right)}{\sum }\text{cost}(u,v)\underset{x\in S\setminus \{u,v\}}{\sum }(\text{cost}(u,x)\\ \;+\;\text{cost}(v,x))\left(\text{TC}(u)+\text{TC}(v)\right)\\ =-\frac{1}{2}\underset{\left\{u,v\}\in \Delta (S\right)}{\sum }\text{cost}(u,v)\left(\text{TC}(u)+\text{TC}(v)\right)\\ \;-\left(2\cdot \text{cost}(u,v)\right)\left(\text{TC}(u)+\text{TC}(v)\right)\\ =-\frac{1}{2}\underset{\left\{u,v\}\in \Delta (S\right)}{\sum }\text{cost}(u,v)\left({\text{TC}}^{2}(u)\right)\\ \;+{\text{TC}}^{2}(v)+2\cdot \text{TC}(u)\cdot \text{TC}(v)\\ \;-\left(2\cdot \text{cost}(u,v)\cdot (\text{TC}(u)+\text{TC}(v))\right)\\ =-\frac{1}{2}\underset{u\in S}{\sum }{\text{TC}}^{3}(u)-\underset{\left\{v,x\}\in \Delta (S\right)}{\sum }\text{cost}(v,x)\cdot \text{TC}(v)\cdot \text{TC}(x)\\ \;+\underset{\left\{y,z\}\in \Delta (S\right)}{\sum }\text{cos}t^{2}(y,z)\left(\text{TC}(y)+\text{TC}(z)\right)\\ =-\frac{1}{2}\underset{u\in S}{\sum }{\text{TC}}^{3}(u)-\underset{e\in E}{\sum }w_{e}\cdot \text{Mult}(e)\\ \;+\underset{u\in S}{\sum }\text{SQ}(u)\cdot \text{TC}(u)\;. \end{array}$$

The next piece that we select from (27) is equal to:

(29) $$\begin{array}{ll} & \;-\frac{1}{2}\underset{\left\{u,v\}\in \Delta (S\right)}{\sum }\text{cost}(u,v)\underset{x\in S\setminus \{u,v\}}{\sum }(\text{cost}(u,x)\;\\ & \;+\text{cost}(v,x))\cdot \text{TC}(x)\;\\ & =-\frac{1}{2}\underset{\left\{u,v\}\in \Delta (S\right)}{\sum }\text{cost}(u,v)(\text{SM}(u)\;\\ & \;+\text{SM}(v)-\text{cost}(u,v)\cdot \text{TC}(u)-\text{cost}(u,v)\cdot \text{TC}(v))\;\\ & =-\frac{1}{2}\underset{u\in S}{\sum }\text{SM}(u)\cdot \text{TC}(u)\;\\ & \;+\frac{1}{2}\underset{\left\{u,v\}\in \Delta (S\right)}{\sum }\text{cos}t^{2}(u,v)\left(\text{TC}(u)+\text{TC}(v)\right)\;\\ & =-\frac{1}{2}\underset{u\in S}{\sum }\text{SM}(u)\cdot \text{TC}(u)+\frac{1}{2}\underset{u\in S}{\sum }\text{SQ}(u)\cdot \text{TC}(u)\;.\; \end{array}$$

For the fourth piece of the sum in (27) we get:

(30) $$\begin{array}{ll} & \;\frac{1}{2}\underset{\left\{u,v\}\in \Delta (S\right)}{\sum }\text{cos}t^{2}(u,v)\underset{x\in S\setminus \{u,v\}}{\sum }\left(\text{cost}(u,x)+\text{cost}(v,x)\right)\;\\ & =\frac{1}{2}\cdot \text{TRS}(B)=\frac{1}{2}\underset{u\in S}{\sum }\text{SQ}(u)\cdot \text{TC}(u)-\text{CB}(T)\;.\; \end{array}$$

The last piece of the sum in (27) can be expressed as:

(31) $$\begin{array}{ll} & \;\frac{1}{2}\underset{\left\{u,v\}\in \Delta (S\right)}{\sum }\text{cost}(u,v)\underset{x\in S\setminus \{u,v\}}{\sum }{\left(\text{cost}(u,x)+\text{cost}(v,x)\right)}^{2}\;\\ & =\frac{1}{2}\underset{\left\{u,v\}\in \Delta (S\right)}{\sum }\text{cost}(u,v)\underset{x\in S\setminus \{u,v\}}{\sum }(\text{cos}t^{2}(u,x)\;\\ & \;+\text{cos}t^{2}(v,x))+3\cdot \text{TRS}(C)\;\\ & =\frac{1}{2}\underset{\left\{u,v\}\in \Delta (S\right)}{\sum }\text{cost}(u,v)(\text{SQ}(u)+\text{SQ}(v)\;\\ & \;-2\cdot \text{cos}t^{2}(u,v))+3\cdot \text{TRS}(C)\;\\ & =\frac{1}{2}\underset{u\in S}{\sum }\text{SQ}(u)\cdot \text{TC}(u)-\text{CB}(T)+3\cdot \text{TRS}(C)\;.\; \end{array}$$

Combining our analyses from (27) up to (31) we get:

$$\begin{array}{lll} \;\text{TRS}(G) & =\frac{1}{2}\cdot \text{TC}(T)\cdot \underset{u\in S}{\sum }{\text{TC}}^{2}(u)\; & \;\\ & \;-\text{TC}(T)\cdot \underset{e\in E}{\sum }w_{e}\cdot \text{TC}(e)-\frac{1}{2}\cdot \underset{u\in S}{\sum }{\text{TC}}^{3}(u)\;\\ & \;-\underset{e\in E}{\sum }w_{e}\cdot \text{Mult}(e)-\frac{1}{2}\cdot \underset{u\in S}{\sum }\text{SM}(u)\cdot \text{TC}(u)\;\\ & \;+\frac{5}{2}\cdot \underset{u\in S}{\sum }\text{SQ}(u)\cdot \text{TC}(u)\;\\ & \;-2\cdot \text{CB}(T)+3\cdot \text{TRS}(C)\;.\; \end{array}$$

We can express TRS(*H*) using the values of the other isomorphism classes:

$$\begin{array}{ll} \;\text{TRS}(H) & =\frac{1}{6}\cdot \left(\underset{\left\{u,v\}\in \Delta (S\right)}{\sum }\underset{\left\{x,y\}\in \Delta (S\right)}{\sum }\underset{\left\{c,d\}\in \Delta (S\right)}{\sum }\text{cost}\left(u,,,v\right)\right)\;\\ & \;\cdot \text{cost}(x,y)\cdot \text{cost}(c,d)-\text{TRS}(A)\;\\ & \;-3\cdot \text{TRS}(B)-6\cdot \text{TRS}(C)-6\cdot \text{TRS}(D)\;\\ & \;-\left(3\cdot \text{TRS}(E)-6\cdot \text{TRS}(F)-6\cdot \text{TRS}(G)\right)\;\\ & =\frac{1}{6}\cdot {\text{TC}}^{3}(T)-\frac{1}{6}\cdot \text{TRS}(A)-\frac{1}{2}\cdot \text{TRS}(B)\;\\ & \;-\text{TRS}(C)-\text{TRS}(D)-\frac{1}{2}\cdot \text{TRS}(E)\;\\ & \;-\text{TRS}(F)-\text{TRS}(G)\;.\; \end{array}$$

We get the value of $E_{R\in \text{Sub}(S,r)}[{\text{MPD}}^{3}(T,R)]$ by plugging into (16) the values that we got for all eight isomorphism classes of triples. For any isomorphism class *X* we showed that the value TRS(*X*) can be computed by using the quantities in Table 1. The lemma follows from the fact that each quantity that appears in this table is used a constant number of times for computing value TRS(*X*) for any class *X*, and since we showed that we can precompute all these quantities in *Θ*(*n*) time in total.

### 

**Theorem 3. ***Let**be a phylogenetic tree that contains s tips, and let r be a natural number with r≤s. The skewness of the mean pairwise distance on**among all subsets of exactly r tips of**can be computed in Θ (n) time.*

### 

*Proof*.According to the definition of skewness, as it is also presented in (1), we need to prove that we can compute in *Θ*(*n*) time the expectation and the variance of the MPD, and the value of the expression $E_{R\in \text{Sub}(S,r)}[{\text{MPD}}^{3}(T,R)]$. In a previous paper we showed that the expectation and the variance of the MPD can be computed in *Θ*(*n*) time. By combining this with Lemma 2 we get the proof of the theorem.

## Experiments: improved *P* value estimation incorporating skewness

Earlier in this paper, we mentioned that distributions of MPD values are often found to be skewed, suggesting that it is necessary to incorporate this skewness into analytical *P* value estimation. However, it is unclear whether good *P* value estimates are possible with only the first three moments of the distribution, or if more detailed distributional information is required.

**Figure 3:**
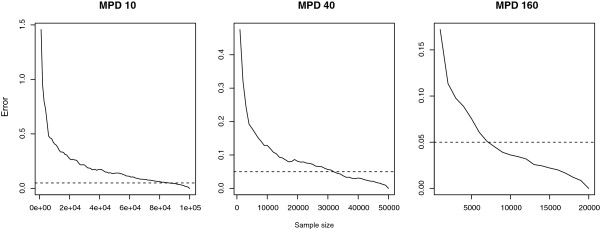
**Error in calculating the distribution that is used as point of reference.** Error in *P* value estimation as the number of tree set resamples increases of tip set sizes of 10, 40 and 160. The dotted lines show errors of 0.05 MPD units, illustrating that the number of resamplings used here were sufficient to estimate percentiles to within 0.05 distance units in each case.

We investigate this question here by considering random phylogenetic trees produced by a pure birth process [12], though results were qualitatively identical when using trees generated by a combined birth-death process (and skewness did not vary as a function of the death rate). We took two approaches for estimating the position of the 2.5 and 97.5 percentile of MPD distribution given a particular tree instance. For any tree  that we constructed, we first calculated the distribution of the MPD values using as a point of reference extensive sampling of sets of tips (much more extensive than is usually employed in practice). In particular, for specific values of *r* we sampled from  a large number of sets that consist of exactly *r* tips (see Table 2 for the values of *r* and numbers of sets that we sampled). We simply calculated the percentiles of these distributions, and call these the *reference* values, recognizing that they neveretheless contain some error, being incomplete samples from the tree. Complete sampling from large trees is computationally infeasible, but we estimate that the error in the calculated percentiles was less than 0.05 distance units in all cases (corresponding to an error of approximately 0.01*%* relative to the mean MPD–see Figure 3).

**Table 2:** The sizes of tip samples that we considered for our experiments, together with the number of sets that we sampled for each tip size in order to derive the “true” values

**Size of each tip sample**	**Number of sampled sets**
10	10^5^
20	10^5^
40	5·10^4^
80	3·10^4^
160	2·10^4^
320	10^4^

**Figure 4:**
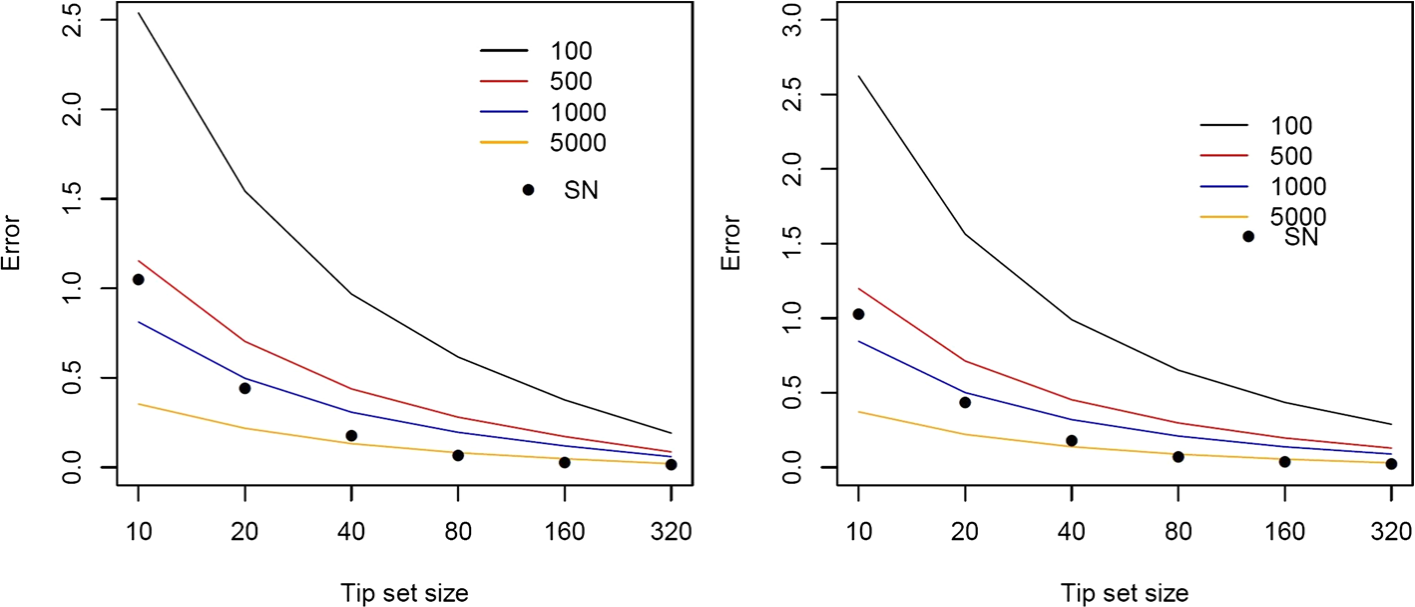
**Comparison of approximation methods.** Errors in *P* value approximation using different resampling replicates (indicated by the coloured lines), compared to that obtained by assuming a skew-normal distribution of MPD values (indicated as SN). Errors were strongly influenced by tip set size *r*, and weakly by tree size; on the left side appear the results for a 500 tip tree, and on the right for a 2000 tip tree). In most cases, *P* value approximation based on the skew-normal distribution performed better than the most commonly-used standard of 1000 set resamplings (blue line), and the relative performance of the skew-normal approach improved with increasing tip set size.

The two approaches that we used to estimate the percentile positions reflect two alternatives that might be employed by practising researchers. In the first approach, for each value *r* that we considered, we sampled again several sets of tips, yet much fewer than the ones we used to calculate the reference values (100, 500, 1000 or 5000 sets). We then compare the absolute difference between percentiles estimated in this manner and the reference values. We refer to this difference as the *error* between the estimated percentile values and the reference values. The second approach uses the mean, variance and skewness of the MPD distribution to determine the position of the 2.5 and 97.5 percentile of the skew-normal distribution with these moments [13]. The mean, variance and skewness were computed in this case based on all the MPD values that we used to calculate reference percentiles. Although we have implemented algorithms for computing the exact values of the mean and variance of the MPD, we have not implemented so far the algorithm that computes the skewness of the MPD; that is the algorithm outlined in the previous sections of this paper. As with the previous approach, the error of this approximation method was calculated by taking the absolute difference between each estimated percentile position and the corresponding reference value.The experiment described above was repeated across 100 replicate trees of each of two sizes (500 and 2000 tips), and across a range of tip set sizes (10, 20, 40, 80, 160 and 320). Errors were weakly related to tree size but decreased strongly with tip set size–see Figure 4. This decrease was more pronounced for estimates based on skew-normal approximation than resampling. Notably, the skew-normal approximation yielded smaller errors than the most commonly used standard of 1000 resamplings for all but the smallest tip set sizes.

Thus, we conclude that the errors introduced by assuming a skew-normal distribution of MPD values appear to be comparable to or smaller than those introduced by standard resampling procedures, while also showing better scaling with increased tip sample size. Finally, the computation of *P* values using skew-normal approximation is typically faster than with resampling, particularly in cases involving large samples of trees.

## Conclusions

Given a rooted tree  and a non-negative integer *r*, we proved that we can compute the skewness of the MPD among all subsets of *r* leaves in  in *O*(*n*) time. An interesting problem for future research would be to implement the algorithm that is outlined by our proof, and show its efficiency in practice. Also, it would be interesting to derive a similar result for the so-called *Community Distance* measure; this is the equivalent of the MPD when distances between two sets of species are considered [11].

## Competing interests

The authors declare that they have no competing interests.

## Authors’ contributions

Both authors have contributed both in developing the research results presented in this paper, as well as in the writing process. Both authors read and approved the final manuscript.
